# Creation of a core outcome set for clinical trials of people with shoulder pain: a study protocol

**DOI:** 10.1186/s13063-017-2054-9

**Published:** 2017-07-20

**Authors:** Joel J. Gagnier, Matthew J. Page, Hsiaomin Huang, Arianne P. Verhagen, Rachelle Buchbinder

**Affiliations:** 1 Department of Orthopaedic Surgery, University of Michigan, MedSport, 24 Frank Lloyd Wright Drive, Lobby A, Ann Arbor, MI 48106 USA; 2 Department of Epidemiology, School of Public Health, University of Michigan, Ann Arbor, MI USA; 3 School of Public Health and Preventive Medicine, Monash University, Melbourne, Australia; 4 School of Social and Community Medicine, University of Bristol, Bristol, UK; 5 Department of General Practice, Erasmus University Medical Center, Rotterdam, Netherlands; 6 Monash University, Melbourne, Australia

**Keywords:** Outcome measures, Core outcome set, Clinical trials, Shoulder, Rotator cuff, Adhesive capsulitis, Osteoarthritis, Pain

## Abstract

**Background:**

The selection of appropriate outcomes or domains is crucial when designing clinical trials, to appreciate the effects of different interventions, pool results, and make valid comparisons between trials. If the findings are to influence policy and practice, then the chosen outcomes need to be relevant and important to key stakeholders, including patients and the public, healthcare professionals and others making decisions about health care. There is a growing recognition that insufficient attention has been paid to the outcomes measured in clinical trials. Recent reviews of the measurement properties of patient-reported outcome measures for shoulder disorders revealed a large selection of diverse measures, many with questionable validity, reliability, and responsiveness. These issues could be addressed through the development and use of an agreed standardized collection of outcomes, known as a core outcome set (COS), which should be measured and reported in all trials of shoulder disorders. The purpose of the present project is to develop and disseminate a COS for clinical trials in shoulder disorders.

**Methods/Design:**

The methods for the COS development will include 3 phases: (1) a comprehensive review of the core domains used in shoulder disorder trials; (2) an international Delphi study involving relevant stakeholders (patients, clinicians, scientists) to define which domains should be core; and (3) an international focus group informed by the evidence identified in phases 1 and 2, to determine which measurement instruments best measure the core domains and identification of any evidence gaps that require further empiric evidence.

**Discussion:**

The aim of the current proposal is to convene several meetings of international experts and patients to develop a COS for clinical trials of shoulder disorders and to develop an implementation strategy to ensure rapid uptake of the core set of outcomes in clinical trials. There would be an expectation that the core set of outcomes would always be collected and reported, but it would not preclude use of additional outcomes in a particular trial.

**Electronic supplementary material:**

The online version of this article (doi:10.1186/s13063-017-2054-9) contains supplementary material, which is available to authorized users.

## Background

The shoulder is one of the more complex joints in the human body and a wide variety of conditions can affect the structures of the shoulder. The most common cause of shoulder pain is rotator cuff disease, which, in one primary care study, accounted for 85% of all shoulder pain presentations [1]. Adhesive capsulitis is also a common cause in middle-aged individuals, while osteoarthritis is becoming increasingly prevalent in older people. Shoulder-related disorders account for substantial medical, economic, and social costs [2, 3]. In 2000, the direct costs for the treatment of shoulder dysfunction in the USA totaled US$7 billion [4, 5]. Nearly 20 million Americans reported shoulder pain in 2005 alone, establishing shoulder pain third only to knee and back pain [6]. A systematic review showed that the estimated prevalence of shoulder pain in the general population varies greatly among studies, with a lifetime reported prevalence ranging from 7% to 67% [7]. With the aging “baby boomer” generation, we can expect the prevalence of shoulder disorders to increase significantly over the next two decades [6].

Shoulder disorders are associated with acute or chronic pain that is often disabling; some disorders also result in weakness and dysfunction of the upper extremity [8]. They have a substantial effect on quality of life, including altered sleep patterns, and adversely impact work and recreation [9]. For example, up to 30% of workers diagnosed with a new episode of shoulder pain take sick leave because of the shoulder disorder [10]. Also, patient-reported outcomes (PROs) research suggests that shoulder disorders may compromise an individual’s health status similar to major medical diseases, including congestive heart failure, acute myocardial infarction, diabetes mellitus, and depression [11, 12].

There are many hundreds of controlled clinical trials for shoulder disorders and some evidence suggests that these studies tend to use a heterogeneous array of outcome measures [13–17]. For example, four recent Cochrane reviews, limited to randomized and quasi-randomized trials investigating manual therapy and exercise or electrotherapy for adhesive capsulitis or rotator cuff disease, included 32, 19, 60, and 47 trials, respectively [13–16]. A review of the included trials found that trialists included a measure of pain in 87%, function in 72%, range of motion in 67%, adverse events in 27%, patient-reported treatment success in 24%, strength in 18%, health-related quality of life in 18%, work disability in 4%, and referral for surgery in 2% [17]. Rotator cuff disease trials more commonly included a measure of strength (26% versus 2% for adhesive capsulitis), whereas adhesive capsulitis trials more commonly included a range of motion measure (82% versus 58% in rotator cuff disease trials). Also, the measurement tools used to assess these domains varied widely. For example, there were 35 different outcome measures for pain. Furthermore, between 1973 and 2014 there was a marked rise in inclusion of a measure of function accompanied by a marked decline in use of a measure of range of movement.

It has been suggested that few outcome measures for shoulder disorders possess acceptable measurement properties (e.g., [18–21]). To be of use for clinical trials and patient care, health status measurement instruments must be valid, reliable, and responsive [22, 23]. A valid tool measures what it proposes to measure and must fulfill requirements for face, content, construct, and/or criterion validity. A reliable instrument measures some phenomenon in a predictable manner (repeatability or reproducibility), whether it is self-reported (test-retest reliability) or is measured by someone else (intra-rater and inter-rater reliability). Finally, a responsive measure is able to detect clinically important change in the underlying construct over time, even if the changes are small, and crucially for clinical trials, must be able to detect clinically important differences in treatment effect [22, 23]. There are several checklists and recommendations on how to assess the full array of psychometric properties across health-status measurement instruments (e.g., [24–27]). Until recently, there was a paucity of studies that have comprehensively assessed outcome measures used for shoulder disorders, or identified where there are gaps in empiric data to guide further research efforts.

A systematic review of PRO measures used in studies of rotator cuff disease identified 73 separate citations for 16 distinct PRO measures [19] and performed a comprehensive assessment of their methodological quality (using the consensus-based standards for the selection of health status measurement instruments (COSMIN) checklist) [24, 25], psychometric properties (using criteria proposed by Terwee et al. [26]), and overall evidence using accepted methods [27]. Outcomes had empiric data supporting an average of only 50% of recommended measurement properties. Tools such as The Western Ontario Rotator Cuff (WORC) Index, Disability of the Arm, Shoulder and Hand measures (DASH), Shoulder Pain and Disability Index (SPADI), and Simple Shoulder Test (SST) had good evidence in support of their measurement properties, while there were concerns about other tools relating to internal consistency, reliability, measurement error, hypothesis testing, and responsiveness.

Another recent systematic review assessed the psychometric properties of shoulder-specific PRO measures using a different tool - Evaluating Measures of Patient Reported Outcomes (EMPRO) [28]. It identified 11 instruments assessed across 112 studies. The American Shoulder and Elbow Surgeons (ASES) shoulder assessment, SST, and Oxford Shoulder Score (OSS) were found to have low administration burden and the best overall scores for validity, reliability, and responsiveness, while the Flexilevel Scale of Shoulder Function, SPADI, and the Dutch Shoulder Disability Questionnaire had some acceptable properties, but several required further evaluation.

A third systematic review used the COSMIN methodological quality checklist to assess questionnaires used to evaluate interventions for rotator cuff disease, including surgery [29]. Sixteen studies evaluating two instruments, the WORC and the Rotator Cuff Quality-of-Life (RC-QOL) measure, were identified. Both tools were found to have adequate construct validity, reliability, responsiveness, internal consistency, and translation but additional methodological aspects - including measurement error, content, structural, cross-cultural and criterion validity, and interpretability - needed further evaluation. A fourth paper assessed the psychometric properties (using criteria proposed by Terwee et al. [26]) of four commonly used shoulder outcome instruments - the ASES, the Constant-Murley score, the DASH and the OSS - and reported that each of them had limited evidence to support their use in shoulder trauma populations [30]. Last, a fifth systematic review of measurement properties of self-administered PRO measures in patients with nonspecific shoulder pain and activity limitations found that none of the seven PRO measures had strong positive evidence for all properties but that the SPADI was the best and was recommended for use in these patients [31].

The lack of uniformity in outcome measurement across trials limits our ability to compare findings between studies or to pool data for meta-analyses. Selective outcome reporting (i.e., selective reporting of favorable or statistically significant outcomes) can also bias the results of systematic reviews [32]. In an effort to reduce heterogeneity in outcomes measured across clinical trials, the development of core outcome sets (COSs) for specific health conditions has been recommended [33]. A COS is defined as an agreed minimum selection of outcomes that should be measured and reported in all clinical trials for a particular health condition [34]. There would be an expectation that the core set of outcomes would always be collected and reported, but it would not preclude use of additional outcomes in a particular trial. A COS would increase the reporting of important outcomes, reduce the risk of selective outcome reporting, and increase the feasibility of conducting meta-analyses on such topics [34, 35]. We searched the COMET database and no COS was identified for this area.

The aim of this project is to develop a COS for clinical trials of shoulder disorders. The Core Outcome Measures in Effectiveness Trials (COMET) [34] and the Outcome Measures in Rheumatology (OMERACT) [36] initiatives provide methodological guidance, including a stepwise approach, for the development of a COS [37, 38]. The long-term goal of this work is to ensure the use of an internationally agreed COS based upon the best available evidence, for all trials of shoulder disorders. This will greatly improve our ability to interpret and compare the findings of different trials and synthesize the evidence in meta-analyses, and will also address the issue of selective outcome reporting.

## Methods

Definitions of key concepts and terms used in this protocol follow those recently outlined by the OMERACT initiative [37] and are presented in Table 1 [39].

**Table 1:** Definitions of concepts [39]

Concept	Definition
Health condition	A situation of impaired health
Health intervention	An activity performed by, for, with, or on behalf of a client(s) the purpose of which is to improve individual or population health, to alter or diagnose the course of a health condition, or to improve functioning
Core area	An aspect of health or a health condition that needs to be measured to appropriately assess the effects of a health intervention (core areas are broad concepts consisting of a number of more specific concepts called domains)
Domain or subdomain	Component of core area: a concept to be measured, a further specification of an aspect of health, categorized within a core area
Outcome	Any identified result in a (sub)domain arising from exposure to a causal factor or a health intervention
Measurement instrument	A tool to measure a quality or quantity of a variable, in this context a (sub)domain or a contextual factor
Outcome measurement instrument	A measurement instrument chosen to assess outcome(s)
Core domain set	For study of health interventions, the minimum set of domains and subdomains necessary to adequately cover all core areas (fully measure all relevant concepts of a specific health condition within a specified scope); it describes what to measure
Core outcome measurement set	The minimum set of outcome measurement instruments that must be administered in each intervention study of a certain health condition within a specified setting to adequately cover a corresponding core domain set; it describes how to measure
Scope	The set of factors that describes the studies and circumstances to which the core outcome set will apply. This is determined by the study questions and includes the health condition(s), target population, interventions, and so forth
Contextual factor	Variable that is not an outcome of the study, but needs to be recognized (and measured) to understand the study results. This includes potential confounders and effect modifiers

This protocol followed recommendations in the SPIRIT checklist (see Additional file 1).

### Establishing a Steering Committee

An International Steering Committee was formed to initiate and support the development of this COS and the Special Interest Group (SIG) at the OMERACT 16 meeting, 11–14 May 2106 in Whistler, BC, Canada. The steering committee includes three individuals with expertise in PRO measures and COS development, including Dr. Rachelle Buchbinder (RB; Australia), Dr. Joel Gagnier (JG; USA) and Dr. Arianne Verhagen (AV; The Netherlands). We also included two fellows, in line with OMERACT recommendations. The project is led by the Project Team (JG, RB), who will coordinate the day-to-day management of the project and meetings. Members of the steering committee and fellows were contacted by email and telephone regarding key decisions.

### Scope of the core outcome set

The COS we develop will apply to measuring efficacy or effectiveness of any health interventions in clinical trials for patients with shoulder disorders. For this COS, shoulder disorders include rotator cuff disorders, adhesive capsulitis, osteoarthritis, instability, dislocation of the shoulder, proximal humeral fractures, and nonspecific shoulder pain. It is expected that all domains of this COS should be included in all clinical trials for shoulder disorders. However, in agreement with the COMET definition [34], this does not imply that primary outcomes of a clinical trial should always be those of the developed COS or that outcome measures should be restricted to the domains of the COS (they can include other domains of relevance to the specific research question).

### Stakeholder involvement

It is routinely recommended that an array of stakeholders be involved in the development of a COS, including scientists/researchers, healthcare providers (e.g., primary care clinicians (including primary care physicians and physical therapists), specialists (e.g., rheumatologists and orthopaedic surgeons, nurses, physician assistants, etc.), patients, government agencies (e.g., funding bodies, healthcare regulators), payers (e.g., healthcare insurance companies, federal health care coverage bodies), and industry representatives [34, 36, 40]. For this COS, the project team will include the following stakeholders:

1. Healthcare scientists/researchers: individuals working in fields of clinical research for shoulder disorders (e.g., orthopaedics, physiotherapy, rheumatology, physical medicine, and rehabilitation), and clinical trial or clinimetric methodologists, or biostatisticians. In addition, any scientists/researchers included as stakeholders in this project will be authors of peer-reviewed scientific articles related to clinical research for shoulder disorders. Healthcare scientists/researchers may also be healthcare providers.
2. Healthcare providers: healthcare professionals from different disciplines (e.g., primary care physicians, physical therapists, rheumatologists, orthopaedic surgeons, nurses, physician assistants), who have clinical experience in the management of patients with shoulder disorders. Healthcare providers may also be healthcare scientists/researchers.
3. Patients: individuals who currently have or have had a shoulder disorder(s), and who sought care for the condition. Some previous research has illustrated that it is limiting not to include patients in the development of a COS [34]. Patients have the perspective of living with the health condition and this may substantially differ from the perspective of researchers and providers.

### A conceptual framework

**Fig. 1:**
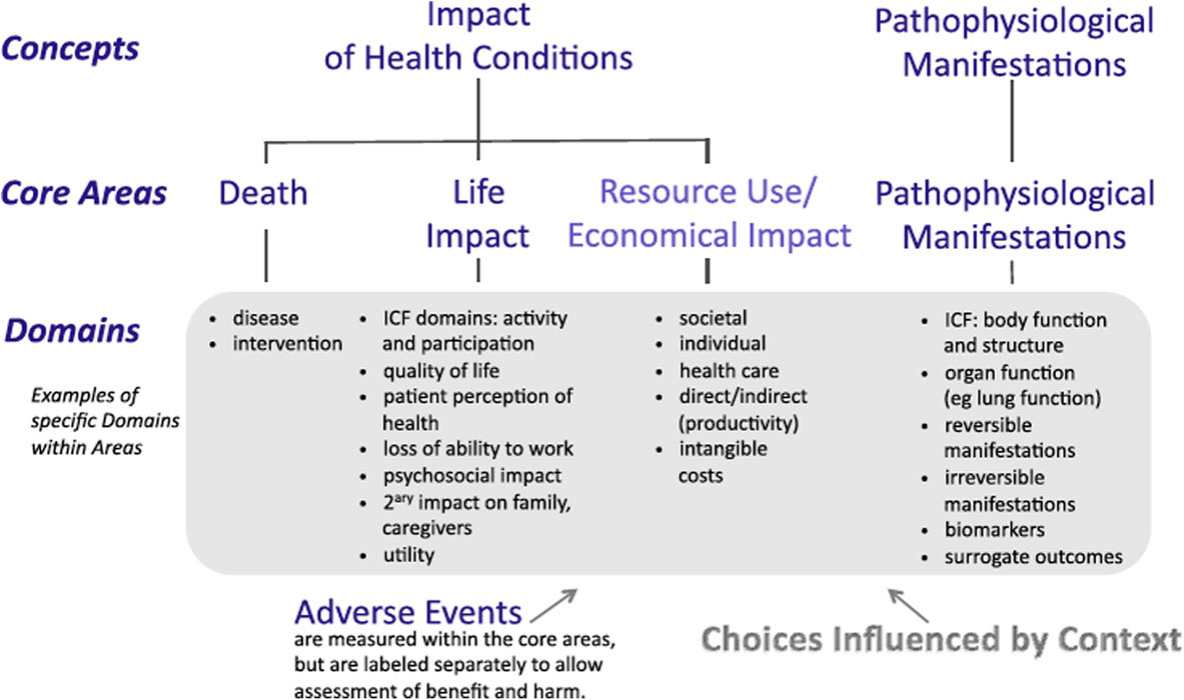
Conceptual framework of core areas for outcome measurement in health intervention studies [38]. *ICF* International Classification of Functioning, Disability and Health

An elucidation of a comprehensive framework of health care is necessary when developing a COS. That is, a COS that focuses on only a few of the components of “health” might be considered narrow. The OMERACT initiative has recently developed a framework that includes key aspects of a health condition to ensure comprehensiveness in COSs (Fig. 1) [38]. This conceptual framework is subdivided into core areas that encompass the complete content of what is measurable in a clinical trial. It includes three core areas that encompass the “impact of health condition” (“death”, “life impact”’, and “resource use/economic impact”) and that describe “pathophysiological manifestations”. Table 2 describes each of these core areas and was adopted from a recently published protocol on the development of COS for nonspecific low back pain [39]. Overall, OMERACT recommends the inclusion of at least one domain from each core area in every COS [37, 38]. The OMERACT Filter 2.0 framework will be used by the Steering Committee to help the development of a list of potential core domains and for discussion during the Delphi study.

**Table 2:** Description of core area outlined in OMERACT Filter 2.0 [33–35]

Core Area	Description
Death	Includes possible specifications of death, such as generic or disease-specific (all-cause versus disease-specific mortality), and intervention-specific (for example, death due to surgery)
Life impact	Can include domains such as activity and participation and domains within the concept of health-related quality of life such as functional status, general health perceptions, and overall quality of life
Resource use/economic impact	Economic impact of health conditions on both society and the individual
Pathophysiological manifestations	This core area is meant to assess whether or not the effect of the intervention specifically targets the pathophysiology of the health condition and may include psychosocial manifestations. Example domains include: body function, reversible manifestations (including modifiable risk factors and actual manifestations of ill health), and irreversible manifestations (including non-modifiable risk factors and damage). This can also include biomarkers and surrogate outcomes

### Research methods

This project will consist of three phases. Phase 1 will generate a list of the core domains. This will include a review of the domains (or subdomains) assessed, or proposed to be assessed, in outcome measures used in clinical research of patients with shoulder disorders. Phase 2 will complete an international Delphi study involving relevant stakeholders (patients, clinicians, researchers/scientists) to define which domains identified in phase 1 should be core. During phase 3 an international focus group informed by the evidence identified in phases 1 and 2 will determine which measurement instruments best measure the core domains and identify any evidence gaps that require further empiric evidence. Phase 3 took place at the OMERACT 2016 meeting. These methods have been used in the development of other COSs (e.g., [39]) and are recommended for use by the OMERACT [37, 38] and COMET initiatives [41]. Finally, we will disseminate our findings to relevant research and scientific groups through publications, conference workshops, meetings and position papers with relevant organizations.

### Phase 1: generation of a list of potential core domains

We will perform a review of health measurement domains and outcome tools used in trials involving people with shoulder disorders. All included trials from relevant Cochrane reviews published in the Cochrane Library will be reviewed. All reviews that synthesize the evidence for the efficacy or effectiveness of any interventions (other than physical therapy) for one the following shoulder conditions will be considered: rotator cuff disease including rotator cuff tears, adhesive capsulitis, osteoarthritis instability, dislocation of the shoulder, proximal humeral fractures and nonspecific shoulder pain. We will use the search strategy for shoulder pain systematic reviews already developed by the Cochrane Musculoskeletal Group. We will also search for and identify randomized controlled trials published within the prior 10 years in PubMed. Two reviewers will assess all studies for inclusion and will extract the domains and outcome tools used in all identified trials, and additional trial characteristics. These domains will then be classified into the core areas described in the OMERACT framework (Fig. 1).

Next, we will invite feedback on these domains from members of the Steering Committee, who will be asked to provide critical comments on each domain. The members will also be asked to indicate if important potential core domains are missing or if certain domains are too broad or should be aggregated. The project team will then finalize the list of potential core domains that will be used during the Delphi study.

### Phase 2: Delphi consensus process

An international Delphi process will be used to reach consensus on the core domains, similar to the process used in another project updating the COS for back pain trials [39]. This method is used to gain consensus among a group of experts or informed respondents that constitute the Delphi panel [34]. The respondents anonymously complete sequential questionnaires that constitute consecutive Delphi rounds. After each round, the group responses are fed back to the panelists who can reconsider their views based on the report of the group views [34]. The Delphi method avoids situations in which the group is dominated by the views of a few prominent personalities.

#### Selection of panel members

On the basis of the findings and methods of related research [39], a minimum of 80 participants will be required in each round of the Delphi. With an expected minimum response rate of 40% [39], at least 200 people will be invited to participate in the Delphi rounds, including an equal number, approximately 67, from each of these three groups: researchers, healthcare providers and patients.

First, a Web of Science search will be performed to compile a list of researchers who have published on one or more shoulder disorders over the last 10+ years (2004–2015). We will also identify and include first and senior authors of a shoulder pain clinical trial or one systematic review.

To identify healthcare providers, Steering Committee members and researchers identified above will be asked to provide the names of clinicians from different disciplines who have experience in managing patients with shoulder disorders.

Next, we will ask Steering Committee members and researchers identified above who have direct contact with patients with shoulder disorders to identify suitable patients to invite to participate. Inclusion criteria for patient participants are as follows: present or past history of shoulder disorders, seeing a healthcare provider for their shoulder pain, and understanding of written English. Patient recruitment should ensure representation across shoulder disorders, so that opinions may represent a wide selection of patient groups, improving the generalizability of our findings. All patient participants, up to a maximum of 70, will be compensated monetarily, US$25, for completion of each voting round, for a total of US$50 each if both rounds are completed.

The final list of all selected panelists will be kept confidential and known only to the project team members.

#### Overview of the Delphi procedure

All panel members will be invited to participate in two Delphi rounds unless they explicitly indicate at some point during the study that they do not wish to receive further invitations. A third round will be conducted only if agreement cannot be reached after two rounds. From the results of phase 1 the project team will develop questions, design the complete Delphi questionnaire, send invitations and reminders to panel members, analyze responses, and formulate feedback reports. We will pilot test the Delphi questionnaires with the Steering Committee members. The online software SurveyMonkey (SurveyMonkey, Palo Alto, CA, USA) will be used to complete the Delphi study. The questionnaire for each round will be available for 4 weeks, with a reminder sent 1 week after the initial invitation [40].

#### Delphi round one

Participants will be given information about the objectives of the study and the questionnaire. First, they will be asked to respond to several demographic questions, including educational and professional backgrounds, country of origin, experience with clinical research relevant to shoulder disorders, and whether they were invited to participate as patients. Next, participants will be asked to rate the importance of the core domains identified in phase 1. Participants will be specifically asked to indicate the importance of each domain to be included in this core domain set; response options will include “Yes”, “No”, and “Unsure/I do not know”. Each question will include an option for comments, and participants will be encouraged to provide a rationale for their ratings and suggest modifications of definitions or wording of the domains. Furthermore, participants will be asked to indicate if there is any overlap between domains, to suggest whether some domains might be aggregated, and to suggest potential core domains not included in the list.

We will calculate frequencies for the response options on the importance of each domain across all participants and for each type of panelist (i.e., patients, clinicians, scientists, clinician-scientists). Responses to open questions will be reviewed and organized thematically to evaluate if significant arguments are against the overall trend in frequencies. Patient responses will be analyzed separately to determine if they differ from the responses of the other panelists. Those domains to which more than two thirds (67%) of the responders choose the response option “Yes” will be included in the list of core domains. Those domains for which there is substantial disagreement across the groups of panelists, or a high percentage (>67%) of “No” responses, will be eliminated from the core set. Those domains with greater than one third but less than two thirds “Ye” responses or some disagreement across the groups of panelists will be reconsidered for voting in round 2. The project team will consider the suggestions for the aggregation of certain domains and the strength of the arguments by deliberation and discussion. Suggested missing core domains will be added to the list for the next Delphi round. All survey findings will be organized into a feedback report. The Delphi survey questions will be modified to reflect these findings for use in round 2.

#### Delphi round two

The feedback report will be emailed to all Delphi participants together with the second round of the survey. Participants will be asked to comment on the feedback report to inform us of any overwhelming and convincing arguments against any of the survey or domain changes. As in round 1, participants will be asked to complete the second round of questions. Specifically, they will be asked to indicate the importance of each domain to be included in this core domain set; response options will include “Yes”, “No”, and “Unsure/I do not know”.

We will calculate frequencies for the response options on the importance of each domain across all participants and for each group of panelists. Responses to open questions will be reviewed to evaluate if significant arguments given are against the overall trend in frequencies. Patient responses will be analyzed separately to determine if they differ from the other panelist responses. All survey findings will be organized into a feedback report. Results will be discussed by the project team, who will assess whether there are substantial arguments against the overall consensus, or to the changes in the survey made from round one and the list of core domains. Finally, if there are clear discrepancies between stakeholder groups or controversial arguments, the results will be presented to the Steering Committee to make final decisions and these finding will be presented during the meetings held during phase 3 of this project.

### Phase 3: focus groups informed by the evidence identified in phases 1 and 2

#### Pre-OMERACT meeting

A pre-OMERACT 16 meeting will be held for all stakeholders with an interest in a COS for shoulder disorders, who will also be invited to participate in the SIG for shoulder disorders at OMERACT 16. We expect that at a minimum, the pre-OMERACT meeting will be attended by the Steering Committee, two fellows, two patient participants, and as many scientists and researchers as possible. All travel, lodging and food-related expenses of the pre-OMERACT focus group attendees will be covered, and each patient participant will receive a US$500 gift.

The purpose of this meeting will be to present the results of the Delphi study and obtain final consensus on the included domains in the shoulder disorder COS. We will present an overview of the rationale for the COS, its purpose, and the process that we have undertaken to develop it. We will then present the consensus on the core domains that should be included.

However, because this process will be driven by evidence-based reviews of what is currently measured in clinical trials, an important aim of the meeting will be to ensure that all domains of importance to the relevant stakeholders have been captured. Therefore, a nominal group process will be conducted to elicit opinion from all attendees and ensure that the full breadth of the burden experienced by patients and observed by experts in the COS domains has been captured. This work will be performed in a fashion similar to that used to develop models for low back pain [42] and osteoarthritis [43]. In both instances, this process identified important aspects of personal burden that are not routinely considered and might be overlooked in a COS [42, 44]. We will also consider whether specific additional domains (and measurement tools of those domains) may be needed for different shoulder disorders (e.g., a measure of strength for rotator cuff disorders and a measure of range of motion for adhesive capsulitis), if these are not part of the core set.

#### Special Interest Group (SIG) meeting at OMERACT 16

At the SIG meeting of the shoulder group at OMERACT 16 we will present our progress to date to all attendees, including the proposed core domain set as determined from the international consensus study and the pre-OMERACT meeting. Attendees will include two patient stakeholders. We will then invite comments and discussion to help refine the core domain set. This will be an important forum to obtain valuable feedback and ensure that the final COS domains receive OMERACT endorsement. We will proceed until the attendees are in agreement with the COS domains, which will be confirmed by a formal vote for or against the domain set.

#### Selection of instruments for COS domains

The Steering Committee and fellows will select outcome measurement instruments that propose to measure the domains identified above. Only instruments that have empirical evidence to support their validity, reliability and responsiveness will be considered. To identify empirical evidence we will search for systematic reviews of instrument properties (e.g., [19]). Where no systematic reviews are available, literature searches will be performed and each instrument assessed using accepted methods [19–27]. All findings will then be organized to arrive at a recommended core outcome measurement set. Several scientists will be consulted for their advice and comments on our choice of instruments.

### Dissemination of the COS recommendations

To hasten the uptake of our findings, we have developed a dissemination plan that considers the intended audience and the best channels of delivery to effect successful implementation of the COS recommendations. In the first instance we will publish our protocol for the formation of the COS. We will then proceed to publish our findings summarizing the core set of domains that should be included in all shoulder trials and subsequently submit for publication the results for which outcome measurement instruments should be used to measure each domain. Our recommendations will be updated on the basis of any further data that might become available in future peer-reviewed publications. Our findings will also be presented at relevant scientific meetings across different disciplines and outcome-measurement-specific venues (e.g., orthopaedics: American Association of Orthopaedic Surgeons (AAOS), Orthopaedic Research Society (ORS) annual meeting, and other orthopaedic meetings internationally that are attended by our working group participants; rheumatology: American College Rheumatology (ACR), the European League Against Rheumatism (EULAR), and Australian Rheumatology Association (ARA) meetings; physical therapy: American Physical Therapy Association (APTA) meeting; COMET annual meeting). Furthermore, we plan to give workshops at several of these same scientific meetings on COS creation for musculoskeletal conditions. We will also publish the recommended COS in the COMET database, where we have already registered two preliminary projects [17, 45].

Importantly we will also evaluate the success of our dissemination strategy on the basis of predetermined short-term and long-term desired outcomes. Examples of the former include the number of times publications that describe the COS are accessed, downloaded, or cited (via citation tracker); examples of long-term desired outcomes would include evidence of use in clinical trials according to trial registry review, and a future review to compare outcomes measured before and after publication of the COS.

## Discussion

The information provided in this protocol is consistent with the information required in the methods section of a full report of a COS, as set out in the COS-STAR guidance [46]. The expected outcome of our project is a core outcome set for use in all clinical trials of shoulder disorders. Use of this core set will markedly improve the value of clinical trials of interventions for shoulder disorders and reduce research waste. It will improve our ability to interpret clinical findings, compare results across different trials and synthesize the best available evidence by meta-analysis. The core outcome set will also reduce the risk of selective outcome reporting. Inclusion of patients, clinicians, and researchers/scientists in the process will ensure that all relevant stakeholder perspectives are captured. Inclusion of recognized international experts who are actively involved in clinical trials in this area in the COS development process will hasten the uptake of the core set into future trials.

## Additional file

Additional file 1: SPIRIT 2013 Checklist: Recommended items to address in a clinical trial protocol and related documents. (DOC 121 kb)

## Abbreviations

ASES: American Shoulder and Elbow Surgeons shoulder assessment
COMET: Core Outcome Measures in Effectiveness Trials
COS: core outcome set
COSMIN: Consensus-based standards for the selection of health status measurement instruments
DASH: Disability of the arm, shoulder and hand measures
OMERACT: Outcome Measures in Rheumatology
OSS: Oxford Shoulder Score
PRO: Patient-reported outcome
SIG: Special Interest Group
SPADI: Shoulder Pain and Disability Index
SST: Simple Shoulder Test
WORC: Western Ontario Rotator Cuff index
